# Optimal strategy of the simultaneous dice game Pig for multiplayers: when reinforcement learning meets game theory

**DOI:** 10.1038/s41598-023-35237-x

**Published:** 2023-05-19

**Authors:** Tian Zhu, Merry Ma, Lu Chen, Zhenhua Liu

**Affiliations:** 1 Department of Applied Mathematics and Statistics, State University of New York at Stony Brook, Stony Brook, NY 11794 USA; 2 Stony Brook School, Stony Brook, NY 11790 USA; 3 Department of Computer Science, State University of New York at Stony Brook, Stony Brook, NY 11794 USA

**Keywords:** Applied mathematics, Computer science, Software, Statistics

## Abstract

In this work, we focus on using reinforcement learning and game theory to solve for the optimal strategies for the dice game Pig, in a novel simultaneous playing setting. First, we derived analytically the optimal strategy for the 2-player simultaneous game using dynamic programming, mixed-strategy Nash equilibrium. At the same time, we proposed a new Stackelberg value iteration framework to approximate the near-optimal pure strategy. Next, we developed the corresponding optimal strategy for the multiplayer independent strategy game numerically. Finally, we presented the Nash equilibrium for simultaneous Pig game with infinite number of players. To help promote the learning of and interest in reinforcement learning, game theory and statistics, we have further implemented a website where users can play both the sequential and simultaneous Pig game against the optimal strategies derived in this work.

## Introduction

Reinforcement learning (RL) serves as an important branch of machine learning. As a powerful approach in decision and control theory, RL has attracted extensive focus, with wide applications in the fields of robotics^1^, quantitative finance^2^, computer vision^3^, healthcare^4^, career planning^5^, gaming^6^ etc. The most common object of a games is to beat the opponents, whether they are computers or other human players. To do so, a player needs to take a sequence of actions as his/her strategy to increase the winning rate and/or decrease the cost (such as time or the number of steps). For some complicated games like Go, despite the great empirical success of Alpha Go based on deep reinforcement learning^7^, the optimal strategy remains unknown. In fact, a new program Alpha Go Zero has outperformed the previous version of Alpha Go known as *Master* after 40 days of self-training by playing millions of games against itself in quick succession and with no input from human players^8^, indicating the current best policy can still be improved. For many simple games, the optimal strategy can be learned via RL algorithms by playing against itself without direct supervision. The dice game Pig is one of them.

The original dice game Pig was publicized by American magician John Scarne in 1945 in his popular book *Scarne on Dice*^9^. This simple one-die game was designed on the sequential basis: Two players take turns to roll a single die until one of them (the winner) reaches a certain goal, which usually is 100 accumulated points. At each turn, a player can keep rolling a die with the scores added to his/her turn total, if no 1 is rolled, or the player wishes to hold so that the turn total will be added to his/her total scores. If a 1 is rolled, however, the player will have all his/her turn total wiped out to zero and it becomes the opponent player’s turn. In summary, during a player’s turn, the player can choose “roll” or “hold”. If the player chooses “roll” and rolls a 1, his/her turn ends automatically with his/her total scores unchanged. If the player chooses “roll” and rolls a number other than 1, namely 2–6, the number will be added to his/her turn total, and the player can choose “roll” or “hold” again. If the player chooses “hold”, the turn total will be added to his/her total scores and his/her turn ends. Pig is known as a “jeopardy dice game”, where previous turn total can be jeopardized by continuing to roll for greater gains (if the next roll is not 1) or ruin (if 1 is rolled next) (Knizia^10^). The optimal strategy to this sequential one-die game is not as simple as the game itself—it has only been solved in recent years using the Markov decision process and dynamic programming (Neller and Presser^11,12^).

Originally the game is played by turns, i.e sequentially, and hence we called it the sequential Pig game. The one-die sequential Pig game has since evolved into several variations, with the most popular one being the two-dice Pig game where two dice are rolled instead of one. The optimal strategies for the standard two-dice sequential Pig game as well as its variation “Double Trouble” have been derived in our previous paper (Zhu and Ma^13^). Apart from increasing the number of the dice rolled, another possible variation is to increase the number of players. The Pig game is originally designed for two players to compete against each other. The game can be easily extended to multiple players without a significant change of the game mechanism. The 3-player sequential Pig game was investigated with the conclusion that the optimal strategy may not exist if the game total points exceed some threshold (Bonnet et al.^14^). As this simple game can be easily played with even more players, say during a party, we are interested in how a certain player will make decisions under such situation. Before we derive the optimal strategy for multiplayers, we first address a potential problem of the current Pig game framework under the multiplayer setting and provide one possible solution.

Both the one-die (Neller and Presser^11^) and the two-dice (Zhu and Ma^13^) sequential Pig games have indicated that the player who plays first has certain advantage in term of the winning probability. The sequential setting is unfair to those played at the end of the playing queue, especially when the number of players increases. Another issue of the sequential game for multiplayer is that if the number of players *n* is large, taking turns to play can decrease each player’s participation rate, which is defined his/her number of turns divided by the sum of the turns that everybody has played, roughly speaking, $\frac{1}{n}$. Keeping one player doing nothing except for watching the others to roll can negatively influence his willingness to participate. To improve the players’ experience and enhance their interest, changing the game playing procedure from sequentially to simultaneously becomes necessary. Like the concepts of sequential game and simultaneous game in game theory, the Pig game is said to be simultaneous if every player begins their own turn at the same time rather than successively. As a result, there is no turn for an individual player but a turn for all players as a whole. A turn ends if all players lose their opportunities to roll. By saying a player loses his opportunity to roll, we mean either of the following two cases happening: (1) a player decides to hold, or (2) a player rolls a 1. If a turn ends without a winner, i.e. nobody reaches the goal, the game will continue and the next turn will begin, with each player’s turn total added to their entire scores respectively. We further assume each play can only see the other players’ turn total at the end of a turn without knowing each individual roll of any players. If multiple players $m \le n$ reach the goal when a turn ends, the reward is defined to be $\frac{1}{m}$ for these *m* winners, and 0 for the other $n-m$ losers. Under this setting, we ensure that the total reward is always 1 regardless of the number of winners.

In this work, we first derive the optimal strategy for the 2-player simultaneous Pig game, including the corresponding optimal strategy against a certain independent strategy, and the optimal mixed-strategy found by the Nash equilibrium, using reinforcement learning and game theory. Meanwhile, we propose a new Stackelberg value iteration for multi-agent (SVIMA) reinforcement learning to derive a near-optimal strategy. Next, we develop the optimal strategy for the 3-player simultaneous game under the independent strategy setting, followed by the extension of the n-player simultaneous game optimal strategy. Subsequently, we discuss the asymptotic behavior of the optimal strategy when *n* goes to infinity. Throughout this paper, the default goal is 100 with the default game setting being simultaneous, unless otherwise stated. We have restricted to the one-die Pig game for this work as well. Finally, to promote the learning of game theory, statistics and reinforcement learning, we have written an online interactive app so that people can play the simultaneous Pig game against our optimal strategies: https://luchencatherine.github.io/pig-game.

## Methods

In this work, we utilize dynamic programming and value iteration by first formulating the game as a Markov Decision Process, and then providing transition probabilities, to find the optimal strategy for the simultaneous dice game Pig. For the fundamental reinforcement learning framework consisting of two main subsections “Markov Decision Process Formulation” and “Dynamic Programming and Value Iteration”, we use the same system of notations and arguments as in our previous work (Zhu and Ma^13^).

### Reinforcement learning framework

#### Markov decision process formulation

The simultaneous Pig game can be solved using reinforcement learning as we are interested in the long-term reward–winning the game. In particular, the game can be viewed as a Markov Decision Process (MDP) as the action is not determined by the past states given the current state. For simplicity, we first consider the 2-player game with player A and his/her opponent player B. The state $s=(i,j)$ contains two elements, player A’s entire score *i*, and the opponent player B’s score *j*, with the state space denoted by *S*. Given such state *s*, player A decides the action *a* he/she should take for this turn and we define *A* to be the action space. For any two states $s,s' \in S$ (not necessarily different) and any action $a \in A$, there is a transition probability $p(s'|s,a)$ that taking action *a* will change the state from *s* to $s'$. The immediate reward on each transition from *s* to $s'$ under action *a* is defined as $r(s,a,s')$ with $r(s,a,s')=\frac{1}{m}$ if $s'$ is a winning state where *m* is the number of winners, and $r(s,a,s')=0$ if $s'$ is a not winning state. The winning state is defined as $i \ge 100$. Because the only situation obtaining the positive reward is winning the game, there is no discount factor needed ($\gamma =1$) to ensure the finite sum of the cumulative reward.

The dice game Pig can be solved using Dynamic Programming (DP) as the exact transition probability from the current state to the next state is known. Given a perfect model, classical DP algorithms can suffer from their great computational costs when the state space is large, which occurs when the number of players increases. However, DP still plays an important role in the field of reinforcement learning as it can be used to compute the exact (optimal) solution in a fully known MDP. Without the complete knowledge of the environment, model-free methods such as Monte Carlo (MC) and Temporal Difference (TD) learning, have less limitations and can often achieve similar effect as DP with less computational expense. These basic model-free methods can be integrated with neural networks to create a new subcategory of the deep reinforcement learning (DRL), with a well-known example being the Deep Q-network (Minh et al.^15^). Recently, many more advanced deep RL methods, such as Double Deep Q-network^16^, Dueling Deep Q-network^17^ and Soft Actor-Critic^18^, have been developed.

#### Dynamic programming and value iteration

Almost all RL algorithms involve the value functions indicating how good a state $v_{\pi }(s)$ is or how good an action is given the state $q_{\pi }(s,a)$, with respect to a policy $\pi$. Once we have found the optimal value functions $v_*(s)$, that satisfy the Bellman optimality equations:

1 $$\begin{aligned} v_*(s) = \max _a \sum _{s'} p(s'|s,a)(r(s,a,s')+v_*(s')), \end{aligned}$$

the optimal policy can be easily obtained by

2 $$\begin{aligned} \pi _*(s)=\mathop {\mathrm{argmax}}_a\sum _{s'} p(s'|s,a)(r(s,a,s')+v_*(s')). \end{aligned}$$

If the dynamics of the environment are perfectly known, we can evaluate a policy $\pi$ by

3 $$\begin{aligned} v_{\pi }(s)={{\,\mathrm{E}\,}}_{\pi }\left[ r(s,a,s')+\gamma v_{\pi }(s')\right] , \end{aligned}$$

which is precisely a system of linear equations. After a policy $\pi$ is evaluated, we can possibly improve the policy through the following greedy update for all state *s*:

4 $$\begin{aligned} \pi '(s)=\mathop {\mathrm{argmax}}_a q_{\pi }(s,a) \end{aligned}$$

Joining policy evaluation and improvement together, we have the policy iteration^19^. Once a policy $\pi$ has been evaluated by the value function $v_\pi$, we can improve the current policy further to generate a better policy $\pi '$ using $v_\pi$. Next, we compute $v_{\pi '}$ and improve it again to yield an even better policy $\pi ''$. Repeat this process until the policy cannot be improved anymore. We then have the sequence of monotonically improved policies and value functions as follows:

5 $$\begin{aligned} \pi _0 \xrightarrow {E} v_{\pi _0} \xrightarrow {I} \pi _1 \xrightarrow {E} v_{\pi _1} \xrightarrow {I} \pi _2 \xrightarrow {E} \cdots \xrightarrow {I} \pi _* \xrightarrow {E} v_* \end{aligned}$$

where $\xrightarrow {E}$ denotes a policy evaluation and $\xrightarrow {I}$ denotes a policy improvement. Because a finite MDP has only a finite number of policies, the convergence to an optimal policy and optimal value function is guaranteed in a finite number of iterations, given the opponent player B’s strategy is independent of player A’s strategy.

For policy iteration, each evaluation $v_\pi$ needs multiple sweeps through the state set to converge in terms of limits. If we wait for exact convergence of every evaluation, the computational cost for policy iteration becomes too high. In fact, policy iteration still converges to the final optimal policy even if the policy evaluation is stopped after only one update of every state. This improved algorithm is termed value iteration^19^.

### Simultaneous one-die Pig game framework

A common heuristic strategy for the dice game Pig is the “hold at n” strategy, which is very simple to use. A player adopting this kind of strategy will continue rolling when his/her turn total is less than *n*, if no 1 is rolled during the turn. If the player’s turn total reaches *n*, the player immediately holds. One exception is when the player needs less than *n* points to reach 100, in which case the player will “hold at 100-*i*” instead with *i* being the player’s entire score. Under the sequential game setting, “hold at 20” in particular, was considered as the best strategy before the optimal strategy was derived by Neller and Presser. As this “hold at n” strategy does not consider the opponent’s score, we refer to this type of strategy by simple strategy in our paper.

Define *p*(*i*, *j*) to be the state-value function, namely the winning probability of the state $s=(i,j)$ at the beginning of a turn, where *i* is player A’s score, and *j* is the opponent player B’s score. Denote the policy that player A follows by $\pi _A$. At the beginning of each turn, player A decides an integer $k=\pi _A(s)=\pi _A(i,j)$ he/she needs to hold at this turn, for example, $k=25$ indicates that player A will hold at 25 for this turn. If the turn total is less than *k*, then player A keeps rolling. As long as player A’s turn total reaches *k* (i.e. greater than or equal to *k*), player A immediately holds. The action is defined as the turn total *k* that player A needs to hold at each given state (*i*, *j*). Obviously, we have the action space $A = \{1,2,\ldots ,100-i\}$. We define *q*(*i*, *j*, *k*) to be the action-value function for the state (*i*, *j*) and the action *k*, where $k\in A$. Then we have:

6 $$\begin{aligned} p(i,j) = \max _k \{q(i,j,k)\}. \end{aligned}$$

For a player deciding to hold at *k*, the turn total he/she will get is not always *k*. Instead, the turn total is a random variable $X_k$, with the set of possible outcomes being $\{0,k,k+1,k+2,k+3,k+4,k+5\}$. The corresponding probabilities are $P_{X_k}(0),P_{X_k}(k),P_{X_k}(k+1),P_{X_k}(k+2),P_{X_k}(k+3),P_{X_k}(k+4),P_{X_k}(k+5)$ respectively, where $P_{X_k}(k)$ is the probability of rolling exactly *k* points this turn (i.e. $P_{X_k}(X_k=k)$). If the game does not end after the turn, then the state at the end of this turn is exactly the state at the beginning of the next turn. Therefore,

7 $$\begin{aligned} \begin{aligned} q(i,j,k) = E_{j'}[p(i,j')P(0)+\sum \limits _{r=0}^5p(i+k+r,j')P_{X_k}(k+r)], \end{aligned} \end{aligned}$$

where $j'$ is the opponent player B’s score after this turn and the expectation is taken with respect to $j'$. Unlike the original sequential Pig game, player A in the simultaneous version knows no information about the opponent player B’s score until the turn ends. The opponent player B has his/her own policy $\pi _B$ with

8 $$\begin{aligned} \pi _B(j,i)=k', \end{aligned}$$

namely the opponent player B decides to hold at $k'$ for given state (*i*, *j*). Then the above Eq. (6) can be further expanded as:

9 $$\begin{aligned} \begin{aligned} q(i,j,k) =&\sum \limits _{r_1=0}^5p(i+k+r_1,j)P_{X_k}(k+r_1)P_{X_{k'}}(0)+\sum \limits _{r_2=0}^5p(i,j+k'+r_2)P_{X_k}(0)P_{X_{k'}}(k'+r_2) \\&+\sum \limits _{r_1=0}^5\sum \limits _{r_2=0}^5p(i+k+r_1,j+k'+r_2)P_{X_k}(k+r_1)P_{X_{k'}}(k'+r_2)+ p(i,j)P_{X_k}(0)_{X_{k'}}P(0) \end{aligned} \end{aligned}$$

Typically the value function *p*(*i*, *j*) is set to be 0 for the terminal states (*i*, *j*) satisfying $i \ge 100$ or $j \ge 100$. If we include the reward in the value function since the intermediate rewards are 0, the terminal values for the 2-player game are:

10 $$\begin{aligned} p(i,j) = {\left\{ \begin{array}{ll} 1,&{} \text { if } i \ge 100 \text { and } j< 100 \\ 0.5,&{} \text { if } i \ge 100 \text { and } j \ge 100 \\ 0,&{} \text { if } i < 100 \text { and } j \ge 100 \\ \end{array}\right. } \end{aligned}$$

#### Distribution calculation

One fundamental issue is how to determine the probability distribution $X_k$ of holding at turn total *k*, i.e. the exactly values of $P_{X_k}(0),P_{X_k}(k),P_{X_k}(k+1),P_{X_k}(k+2),P_{X_k}(k+3),P_{X_k}(k+4),P_{X_k}(k+5)\}$. We start from the simplest case $X_1$. If a player holds at 1, (s)he will get one of the points from the outcome set $O=\{0,2,3,4,5,6\}$, since when (s)he rolls a 1, according to the rule, (s)he will get 0 turn total and her/his turn ends. The probability of each outcome is trivial, $\frac{1}{6}$. To be consistent with our previous notation, we extend the outcome set to be $O=\{0,1,2,3,4,5,6\}$ with additional probability $P_{X_1}(1)=0$. For $X_2$, obviously the outcome set is $O=\{0,2,3,4,5,6\}$ with each probability being $\frac{1}{6}$. Again, we extend the outcome set to be $O=\{0,2,3,4,5,6,7\}$ with probability $P_{X_2}(7)=0$ to satisfy the general $\{P_{X_k}(0),P_{X_k}(k),P_{X_k}(k+1),P_{X_k}(k+2),P_{X_k}(k+3),P_{X_k}(k+4),P_{X_k}(k+5)\}$ notation.

In general, we can recursively compute $P_{X_{k+1}}$ utilizing $P_{X_k}$. The range of $X_{k+1}$ is $\{0,k+1,k+2,k+3,k+4,k+5,k+6\}$, with the first 6 numbers overlapping with the range of $X_k$. The number of rolls for $X_{k+1}$ must be greater than or equal to that of $X_k$. If the numbers of rolls are equal, we have $X_k \in \{0,k+1,k+2,k+3,k+4,k+5\}$. If the numbers of rolls are not equal, i.e. one more roll is needed, we know $X_k=k$. For the next roll, face 1,2,3,4,5,6 are equally likely, corresponding to the turn total $0,k+2,k+3,k+4,k+5,k+6$, with each probability being $\frac{1}{6}$. Therefore, we have the following recursive relationship (also summarized in Table 1):

11 $$\begin{aligned} P_{X_{k+1}}(i) = {\left\{ \begin{array}{ll} P_{X_k}(i)+\frac{1}{6}P_{X_k}(k),&{} \text {if } i \in \{0,k+2,k+3,k+4,k+5\} \\ P_{X_k}(i),&{} \text {if } i=k+1\\ \frac{1}{6}P_{X_k}(i),&{} \text {if } i=k+6 \end{array}\right. } \end{aligned}$$

**Table 1:** The relationship between the probability distribution of $X_k$ (hold at k) and the probability distribution of $X_{k+1}$ (hold at k+1).

Prob.	0	k	k+1	k+2	k+3	k+4	k+5	k+6
$X_k$	$p_1$	$p_2$	$p_3$	$p_4$	$p_5$	$p_6$	$p_7$	0
$X_{k+1}$	$p_1+\frac{p_2}{6}$	0	$p_3$	$p_4+\frac{p_2}{6}$	$p_5+\frac{p_2}{6}$	$p_6+\frac{p_2}{6}$	$p_7+\frac{p_2}{6}$	$\frac{p_2}{6}$

An example is to see the probability distribution of $X_3$ using the distribution of $X_2$. The range of $X_3$ is $\{0,3,4,5,6,7,8\}$. If the first roll is not 2, then the player immediately stops (holds or passively stops for rolling a 1). If the first roll is 2, the player needs to roll again, and the new turn total is one of 0, 4, 5, 6, 7, 8 with each probability being $\frac{1}{6}P_{X_2}(2)$. Thus,

$$\begin{aligned} P_{X_3}(i) = {\left\{ \begin{array}{ll} P_{X_2}(i)+\frac{1}{6}P_{X_2}(2) = \frac{7}{36},&{} \text {if } i \in \{0,4,5,6\} \\ P_{X_2}(i)+\frac{1}{6}P_{X_2}(2) = \frac{1}{36},&{} \text {if } i = 7\\ P_{X_2}(i) = \frac{1}{6},&{} \text {if } i=3\\ \frac{1}{6}P_{X_2}(i) = \frac{1}{36},&{} \text {if } i=8 \end{array}\right. } \end{aligned}$$

As mentioned earlier, we set $P_{X_2}(7)=0$, rendering one more equation in this example. The numerical results of some distributions of interest are listed in Table 2.

**Table 2:** Detailed probability distributions for $X_{20},X_{21},X_{22},X_{23},X_{24},X_{25},X_{100}$.

Prob.	0	k	k+1	k+2	k+3	k+4	k+5
$X_{20}$	0.6245	0.0997	0.0950	0.0742	0.0542	0.0352	0.0172
$X_{21}$	0.6412	0.0950	0.0908	0.0708	0.0518	0.0338	0.0166
$X_{22}$	0.6570	0.0908	0.0866	0.0676	0.0496	0.0325	0.0158
$X_{23}$	0.6721	0.0866	0.0828	0.0648	0.0476	0.0310	0.0151
$X_{24}$	0.6866	0.0828	0.0792	0.0620	0.0454	0.0296	0.0144
$X_{25}$	0.7004	0.0792	0.0758	0.0592	0.0434	0.0282	0.0138
$X_{100}$	0.9898	0.0027	0.0026	0.0020	0.0015	0.0010	0.0005

The range $\{0,k,k+1,k+2,k+3,k+4,k+5\}$ is not the same for all variables. Instead, it depends on the random variable $X_k$. For instance, the range of $X_{20}$ is $\{0,20,21,22,23,24,25\}$ while the range of $X_{25}$ is $\{0,25,26,27,28,29,30\}$.

## Results

### Optimal strategy for 2-player simultaneous one dice game Pig

#### Optimal strategy given independent opponent strategy

We first derive the optimal strategy for player A given the opponent player B’s strategy is independent of A. By independent, we mean that the opponent player B’s strategy will not change if player A’s strategy changes. The optimal strategy is learned by the agent itself without any direct supervision.

With the independence strategy assumption, the corresponding optimal strategy $\pi _{A*}$ for player A can be found by Algorithm 0.1, given a certain strategy $\pi _B$ for the opponent player B. One example is to let the opponent B use the simple “hold at n” strategy. One question is what *n* is the best choice? We compare the winning rate of the corresponding optimal strategy found by Algorithm 0.1 against the “hold at n” strategy with different reasonable selections of *n*, as Fig. 1 shows, with the detailed numbers shown in Supplementary Table S1. Figure 1 indicates that $n=25$ is the best scenario as the winning probability of the corresponding optimal strategy is the lowest among all these choices of *n*, but the winning rate is still above 50%. One interesting fact is that there are several local minimums, namely $n=16,20,25,33$, which are exactly $\frac{100}{6},\frac{100}{5},\frac{100}{4},\frac{100}{3}$. These strategies have special meanings, i.e. to win the game in 6,5,4,3 turns respectively.

**Figure 2:**
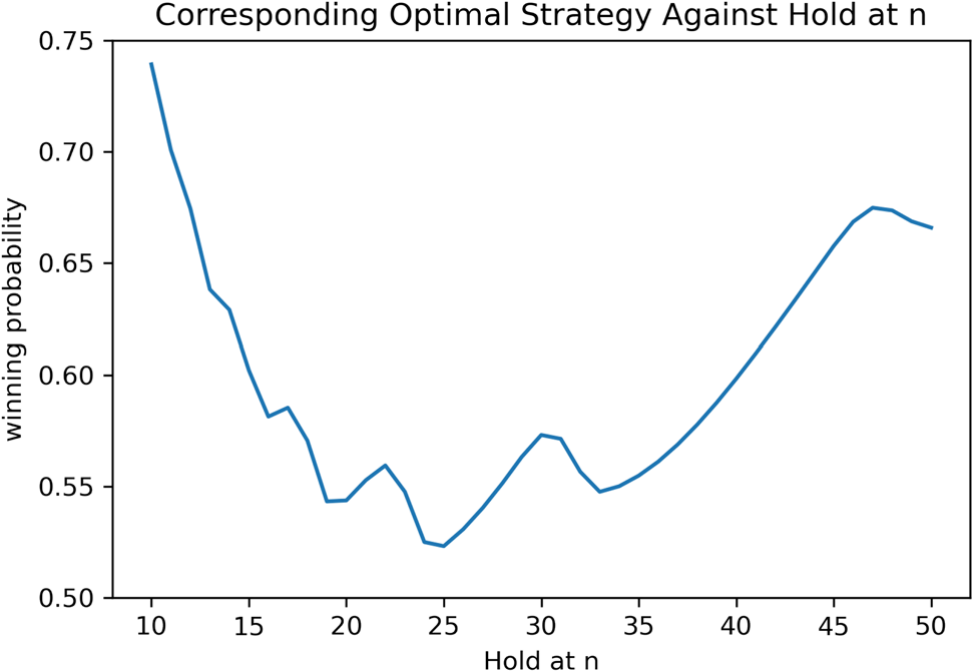
The winning probabilities of the corresponding optimal strategy against the “hold at n” strategy. The *x*-axis is the choice of *n*.

**Figure 3:**
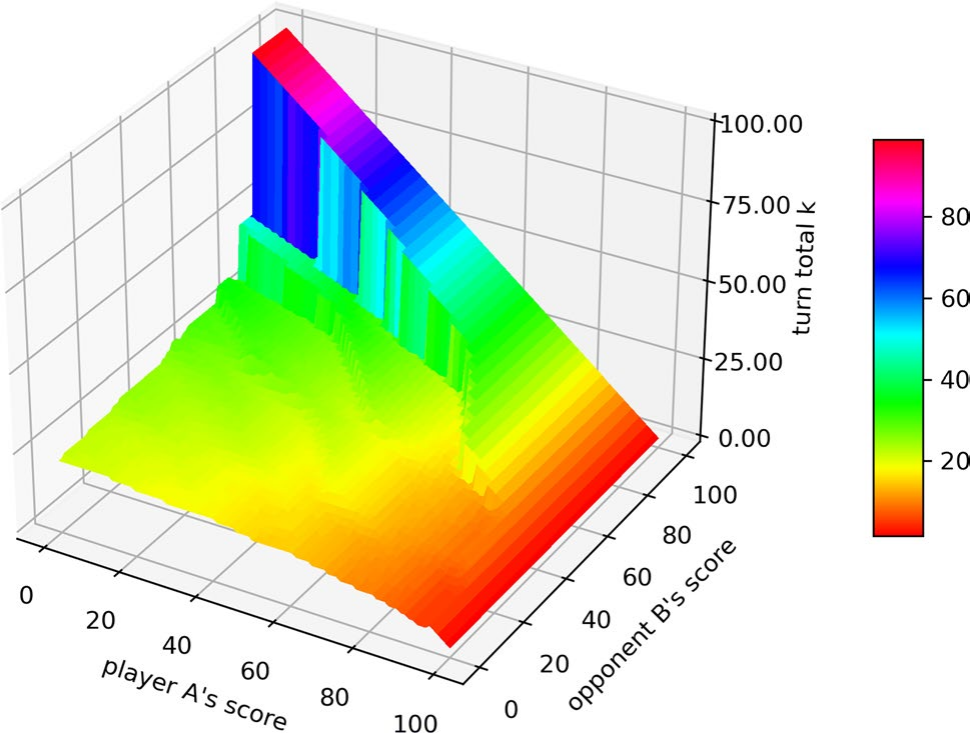
The contour surface of the decisions, i.e. the value *k* that player A should hold at for a given state, obtained from the corresponding optimal strategy against the “hold at 25” strategy.

**Figure 4:**
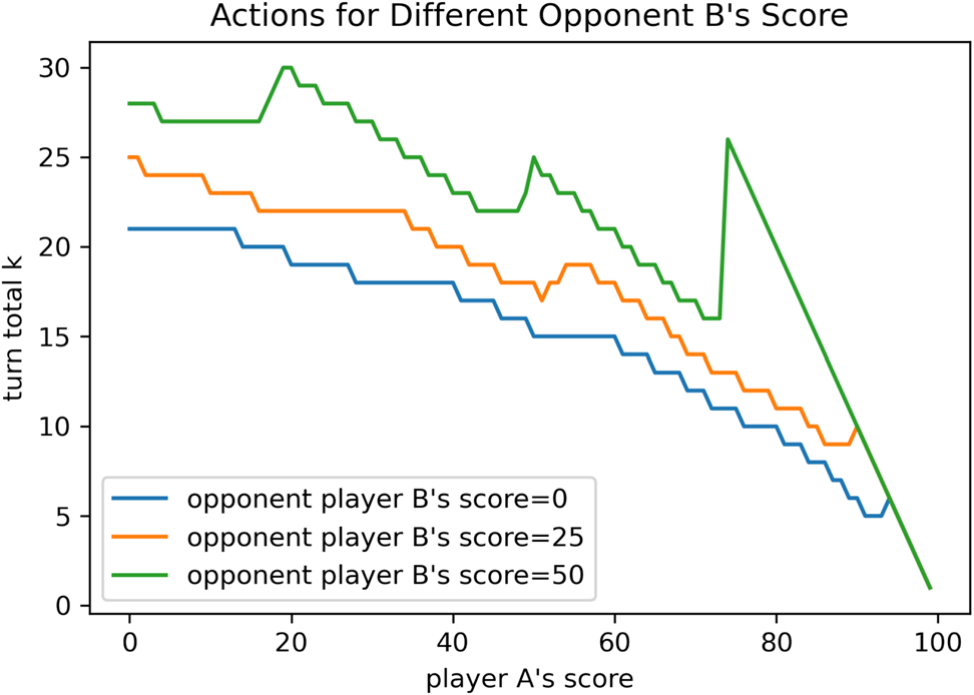
The actions for player A given different opponent’s scores, when the opponent player B uses the “hold at 25” strategy.

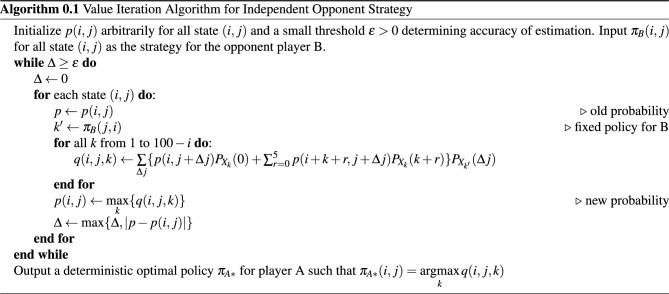



For $n=25$, i.e. against the “hold at 25” strategy, the decision from the corresponding optimal strategy is shown in Fig. 2, from which we conclude that the corresponding optimal strategy acts increasingly more aggressively as the opponent player B’s score approaches 100, especially when player A’s score is relatively low. In specific, when the opponent player B’s score is fixed, the action (turn total *k*) player A will take versus his/her own score is plotted in Fig. 3. There are some “jump points” which show the significant changes in actions when player A’s score only perturbs a little. This phenomenon is more obvious when the opponent player B’s score is closer to 100. This fact suggests that when the opponent is getting closer to win, instead of recklessly play aggressively, player A should increase his/her aggressiveness step by step, after his/her score exceeds some thresholds.

The discontinuities are caused by the following two facts:

1. Given the opponent’s score is fixed, the turn total a player would hold at should gradually decrease when his/her own score increases, to avoid the risk of losing all turn total.
2. Given the opponent’s score is fixed (especially large), the turn total a player would hold at should increase at some points. As the player’s score pass some threshold, i.e., closer to the goal (i.e., 100), (s)he should take the risk of rolling higher turn total to increase the winning probability. Those points are typically around 50 and 75, as they are usually the threshold to reduce the number of turns needed to win the game by 1 (on average a player should hold at around 25).

Therefore, those “jumps” can be viewed as the trade-off between the profit (possibly reducing the number of turns needed) and the risk (possibly losing all turn total). Those discontinuities demonstrate the power of reinforcement learning because human strategies such as “hold at 25” or “keep pace” can never detect those change points.

One remark is that, with the independence assumption, the opponent player B’s strategy can be a more complex pure strategy, i.e. a spline function of player A’s entire score, instead of a simple “hold at n” strategy. An example is player B will hold at 25 if player A’s score is less than 40, hold at 30 if player A’s score is between 40 and 60, and hold at 100-*j* (where *j* is player B’s entire score at the beginning of the turn) if player A’s score is greater than 60. Moreover, the opponent player B can even utilize randomness so that his/her strategy will become a mixed strategy. The algorithm still works in such case with some slight modifications. Since now

$$\begin{aligned} \pi _{B}(i,j) = k_l \text { with probability } p_{k_{l}}, \end{aligned}$$

we adjust the action-state value *q*(*i*, *j*, *k*) of the state (*i*, *j*) and the action *k* for player A according to the following:

12 $$\begin{aligned} q(i,j,k) = \sum \limits _{l}\left\{ \sum \limits _{\Delta j}\left\{ p(i,j+\Delta j)P_{X_k}(0)+\sum _{r=0}^5 p(i+k+r,j+\Delta j)P_{X_k}(k+r)\right\} P_{X_{k_l}}(\Delta j)\right\} p_{k_l} \end{aligned}$$

#### Optimality of value iteration for independent opponent strategy

Denote the old policy by $\pi$. For each iteration, we find a new policy $\pi '$ through maximizing the action-value function *q*, i.e. $\pi '(i,j)=\mathop {\mathrm{argmax}}_k q_{\pi }(i,j,k)$, such that $p_{\pi '}(s) \ge p_{\pi }(s)$ for the given state $s=(i,j)$. For another state $s'$ satisfying $s' \ne s$, we have $p_{\pi '}(s') = p_{\pi }(s')$ by the independence assumption. Thus, the new policy $\pi '$ must be as good as or better than the old policy $\pi$ since $p_{\pi '}(s) \ge p_{\pi }(s)$ for any state *s*. Suppose the value iteration converges, so the new greedy policy $\pi '$, is as good as, but not better than, the old policy $\pi$. Then $p_{\pi '}=p_{\pi }$ and for all state *s*, it follows that:

13 $$\begin{aligned} \begin{aligned} p_{\pi '}(s)&= {{\,\mathrm{E}\,}}\left[ R_{t+1}+\gamma p_{\pi }(S_{t+1})|S_t=s,A_t=\pi '(s)\right] \\&= {{\,\mathrm{E}\,}}\left[ R_{t+1}+\gamma p_{\pi }(S_{t+1})|S_t=s,A_t=\mathop {\mathrm{argmax}}\limits _k q_{\pi }(i,j,k)\right] \\&= \mathop {\max }\limits _a {{\,\mathrm{E}\,}}\left[ R_{t+1}+\gamma p_{\pi }(S_{t+1})|S_t = s,A_t=a \right] \\&= \mathop {\max }\limits _a {{\,\mathrm{E}\,}}\left[ R_{t+1}+\gamma p_{\pi '}(S_{t+1})|S_t = s,A_t=a \right] \\&= \mathop {\max }\limits _a \mathop {\sum }\limits _{s',r} P(s'|s,a)(r+\gamma p_{\pi '}(s')) \end{aligned} \end{aligned}$$

which is exactly the Bellman optimality equation. Therefore, $p_{\pi '}$ must be $p_*$, and both $\pi$ and $\pi '$ must equal to the optimal policy $\pi _*$, which completes the optimality of the solution found by value iteration.

#### Mixed-strategy equilibrium

If both players play rationally, i.e. assuming player A and the opponent player B adopt the same strategy, then for all state (*i*, *j*) we have

14 $$\begin{aligned} \pi _A(i,j)=\pi _B(i,j), \end{aligned}$$

and the solution (if exists) in the above system (12) together with Eqs. (5)–(8) is the optimal policy $\pi _*$, satisfying the Nash equilibrium. Unfortunately, the value iteration does not converge under this setting due to the independence assumption being violated. The iteration leads to a cycle of policy changes of holding at different numbers given the same state, with $\pi _{l_1}$ and $\pi _{l_2}$ being two cyclic policies:

$$\begin{aligned} \pi _0 \xrightarrow {} \pi _1 \xrightarrow {} \pi _2 \xrightarrow {} \cdots \xrightarrow {} \pi _{l_1} \xrightarrow {} \pi _{l_2} \xrightarrow {} \pi _{l_1} \xrightarrow {} \pi _{l_2} \xrightarrow {} \cdots \end{aligned}$$

For example, $\pi _{l_1}(47,69)=16$ and $\pi _{l_1}(69,47)=26$. To compute $\pi _{l_2}(47,69)$ for player A, the previous information about the state (69, 47), namely $\pi _{l_1}(69,47)$, is required as it serves as the action for the opponent player B. Given $\pi _{l_1}(69,47)=26$, the new policy $\pi _{l_2}$ at the state (47, 69) is $\pi _{l_2}(47,69)=28$. To compute $\pi _{l_2}(69,47)$, we use the information $\pi _{l_2}(47,69)=28$, rendering $\pi _{l_2}(69,47)=31$. When the next time value iteration encounters the state (47, 69), using $\pi _{l_2}(69,47)=31$, the new policy $\pi _{l_3}$ at (47, 69) is 16. Following the same logic, the update for (69, 47) is $\pi _{l_3}(69,47)=26$, which is exactly $\pi _{l_1}(69,47)$. Except for the 141 states involving cyclic changes, the policy values of the rest 9859 states remain the same after 60 iterations.

Since $\pi _{l_1}$ and $\pi _{l_2}$ alternate, it is hard to determine which policy is better than the other. For state (0, 0) which is at beginning of the game, the winning probability of one against the other is very close to 0.5, with error less than $10^{-6}$. The mean absolute error (MAE) of these two policies $\pi _{l_1}$ and $\pi _{l_2}$ over all states is: $\frac{1}{10000}\sum \limits _{i=0}^{99} \sum \limits _{j=0}^{99} |p_{\pi _{l_1}}(i,j) - p_{\pi _{l_2}}(i,j)| = 3.346\times 10^{-7}$, with the maximum error being $p_{\pi _{l_1}}(69,47) - p_{\pi _{l_2}}(69,47) = 2.020 \times 10^{-4}$. Another interesting fact is that if we use either $\pi _{l_1}$ or $\pi _{l_2}$ against “hold at 25” strategy, which is the best among “hold at n” class in simultaneous one-die Pig game, the winning rate is 0.5192, while the winning rate of the corresponding optimal strategy against “hold at 25” is 0.5231 as we mentioned in the previous subsection.

Even the game is simultaneous, the policy update does have order. Here we update the policy $\pi _A$ for player A and then force the policy $\pi _B$ for the opponent player B to be equal to $\pi _A$. We can view this process as player A actively adjust his/her policy while the opponent player B passively follows player A’s strategy. We define the payoff for a player being 1 if his/her strategy is “preferable” by value iteration, and the payoff being 0 otherwise. If both players adopt the same strategy, the winning probability for either player will be 0.5 due to symmetry. Thus, both players’ payoff will be 0.5 if they adopt the same strategy. Here, we say a policy $\pi '$ is preferable by value iteration than another policy $\pi$ if value iteration will update the current policy $\pi$ with a new policy $\pi '$. The payoff matrix then can be constructed as shown in Table 3:

**Table 3:** Payoff for the simultaneous one-die Pig game with two players (player A, player B).

		Player *B*	
		Policy $\pi _{l_1}$	Policy $\pi _{l_2}$
Player *A*	Policy $\pi _{l_1}$	(0.5, 0.5)	(1, 0)
	Policy $\pi _{l_2}$	(1, 0)	(0.5, 0.5)

The pure-strategy equilibrium does not exist in this game, because neither player A nor the opponent player B would deviate from any profile of strategies. For example, $(\pi _{l_1},\pi _{l_1})$ is not an equilibrium because player A can switch the policy to $\pi _{l_2}$ to increase his/her payoff from 0.5 to 1. $(\pi _{l_2},\pi _{l_1})$ is either not an equilibrium because player B can deviate his/her strategy to $\pi _{l_2}$ and player B’s payoff increases from 0 to 0.5.

Due to the fact that every finite game has a Nash equilibrium (Nash^20^), we can find the mixed-strategy Nash equilibrium of this game. A mixed-strategy combines each pure strategy stochastically with fixed probability. The equilibrium has the property that the payoffs from selecting the policy $\pi _{l_1}$ and $\pi _{l_2}$ are exactly the same, for both players. Assuming player B adopts policy $\pi _{l_1}$ with probability *b*, so player B has $(1-b)$ probability selecting policy $\pi _{l_2}$. From player A’s point of view, the expected payoff for selecting policy $\pi _{l_1}$ is: $b \times 0.5 + (1-b) \times 1 = 1-0.5b$; meanwhile, the expected payoff for selecting policy $\pi _{l_2}$ is: $b \times 1 + (1-b) \times 0.5 = 0.5+0.5b$. Equating these two quantities together, we have $b=0.5$. Similarly, if we assume player A selects policy $\pi _{l_1}$ with probability *a*, player A will select policy $\pi _{l_2}$ with probability $(1-a)$. Equating player B’s expected payoffs from selecting policy $\pi _{l_1}$ and $\pi _{l_2}$, we have $a \times 0.5 + (1-a) \times 0 = a \times 0 + (1-a) \times 0.5$, rendering $a=0.5$. Therefore, the mixed-strategy equilibrium is $(P_{\pi _A}(\pi _{l_1})=0.5,P_{\pi _B}(\pi _{l_1})=0.5)$. Notice that at the equilibrium, the policy $\pi _A$ for player A and the policy $\pi _B$ for player B are the same, which is the optimal strategy $\pi _*$ for the simultaneous 2-player Pig game.

#### Stackelberg value iteration for multi-agent reinforcement learning

For two players, the divergence is caused by forcing to set the two players’ policies to be the same while one policy is changed slightly later than the other, like “chasing one’s own tail”. However, this loop can be avoided under the multi-agent reinforcement learning setting, from which we developed a Stackelberg Value Iteration for multi-agent reinforcement learning (SVIMA). For multi-agent reinforcement learning, Mcmahan (2003) proposed the Double Oracle Algorithm (DOA) based on game theory^21^, and later Lanctot (2007) developed DOA’s generalization form Policy-Space Response Oracle (PSRO)^22^. While both our SVIMA and DOA involve the minimax operator, SVIMA can be viewed as a generalization of value iteration for multi-agent learning which alternatives evaluate all possible actions and update the value function. For DOA and PSRO, the policy set is restricted at the beginning and the newly found policy is continuously added to the policy set until convergence. As the nature of dynamic programming, SVIMA relies on the full information on the transitions between each state. Thus, SVIMA does not require simulations, unlike PSRO which usually depends on fictious play where a payoff table (or the winning probabilities) is updated through the simulated observed outcomes, as mentioned in one of its application (Muller^23^).

Under the multi-agent RL setting, in specific, for a Markov game, there is a collection of actions sets $A_1,A_2,\ldots ,A_k$, one for each agent in the environment. States are evolved through an action from each agent based on the current state: $S \times A_1 \times A_2 \times \cdots \times A_k \rightarrow S$. Each individual agent *i* has one reward function $R_i: S \times A_1 \times A_2 \times \cdots \times A_k \rightarrow R$. The goal of each individual agent is to maximize his/her own cumulative discounted reward, under the assumption of no collusion (Littman^24^).

In our simultaneous dice game for two players, we let player A and the opponent player B be two individual agent and they would compete against each other. We use the Stackelberg model to find the subgame perfect Nash equilibrium (SPNE). The Stackelberg competition model was first developed in economics, with its application in many fields, such as system control^25^. Considering the nature of a simultaneous game, instead of the Stackelberg competition model where the leader usually has substantial advantage, the Cournot model is more appropriate. However, the actions for some states are saddle points if applying the Cournot model, as the solution space is a subset of integers. The results are consistent with our fore-mentioned mixed-strategy. Under the simultanoues dice game settings, these two players are exactly identical in terms of information and winning rates. Consequently, applying the Stackelberg model to our problem will produce a very close approximation to the exact solution found by the Cournot model, according to a weak version of first-mover’s equilibrium profit (Osborne^26^).

For two policies $\pi$ and $\pi '$, the winning probability for player A is $E_{X_{\pi },X_{\pi '}}[p(i+X_{\pi (i,j)},j+X_{\pi '(j,i)})]$, using the same notation $X_k$ defined above, which is a random variable denoting the turn total under “hold at k”. For player A, (s)he wants to maximize the above quantity. While for player B, (s)he wants to maximize $1-E_{X_{\pi },X_{\pi '}}[p(i+X_{\pi (i,j)},j+X_{\pi '(j,i)})]$ as the total winning probabilities sum up to 1. Equivalently, player B wants to minimize $E_{X_{\pi },X_{\pi '}}\left[ p(i+X_{\pi (i,j)},j+X_{\pi '(j,i)})\right]$. Therefore, the Stackelberg equilibrium for each state (*i*, *j*) can be found by solving the following system:

15 $$\begin{aligned} {\left\{ \begin{array}{ll} \frac{\partial E_{X_{\pi },X_{\pi '}}\left[ p(i+X_{\pi (i,j)},j+X_{\pi '(j,i)})\right] }{\partial \pi } = 0 \\ \frac{\partial E_{X_{\pi },X_{\pi '}}\left[ p(i+X_{\pi (i,j)},j+X_{\pi '(j,i)})\right] }{\partial \pi '} = 0 \end{array}\right. } \end{aligned}$$

The idea behind SVIMA is that given the opponent’s strategy $\pi '$, player A can easily find an optimal policy $\pi _A$ against the opponent, through maximizing one’s own winning probability.

16 $$\begin{aligned} \pi _A(i,j) = \mathop {\mathrm{argmax}}_{\pi } E_{X_{\pi },X_{\pi '}}\left[ p_A(i+X_{\pi (i,j)},j+X_{\pi '(j,i)})\right] =f(\pi ')(i,j) \end{aligned}$$

The problem then reduces to determining how the opponent will choose his/her policy $\pi _B$. Using the fact that $\pi _A$ is a function of $\pi '$, the opponent player can choose a policy $\pi '$ that among all $\pi '$s, the winning probability for player A is minimized as the game is zero-sum,

17 $$\begin{aligned} \pi _B(i,j) = \mathop {\mathrm{argmin}}_{\pi '} E_{X_{\pi _A},X_{\pi '}}\left[ p_A(i+X_{\pi _A(i,j)},j+X_{\pi '(j,i)})\right] = \mathop {\mathrm{argmin}}_{\pi '} E_{X_{f(\pi ')},X_{\pi '}}\left[ p_A(i+X_{f(\pi ')(i,j)},j+X_{\pi '(j,i)})\right] \end{aligned}$$

Below is the detailed algorithm 0.2 for SVIMA for our simultaneous two-player Pig game. The policies outputted by the algorithm is shown in Fig. 4. The winning rates for two players at the beginning of the game are 0.4999997 and 0.5000003, respectively, very close to the theoretical results, namely 0.5 and 0.5. Due to the discretization approximation and numerical errors, the two policies $\pi _A$ and $\pi _B$ are not exactly the same, with 74 out of total 10,000 states different, so we call it the near-optimal policy. The actions, however, are all differed by 1 for different actions. The detailed difference is listed in Supplementary Table S3. Another fact demonstrating the near-optimal property is that the winning probability of the Stackelberg strategy against the fore-mentioned mixed-strategy is also very close to 0.5, with error less than $10^{-6}$.
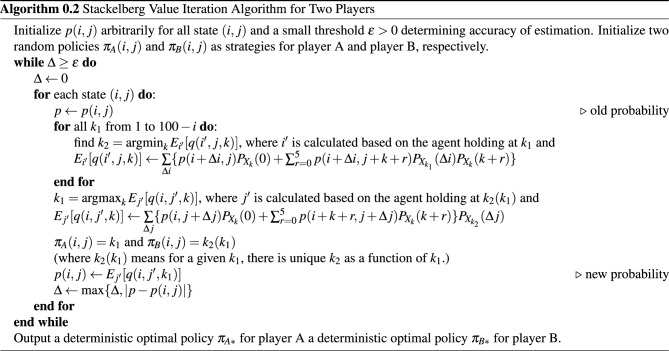

**Figure 5:**
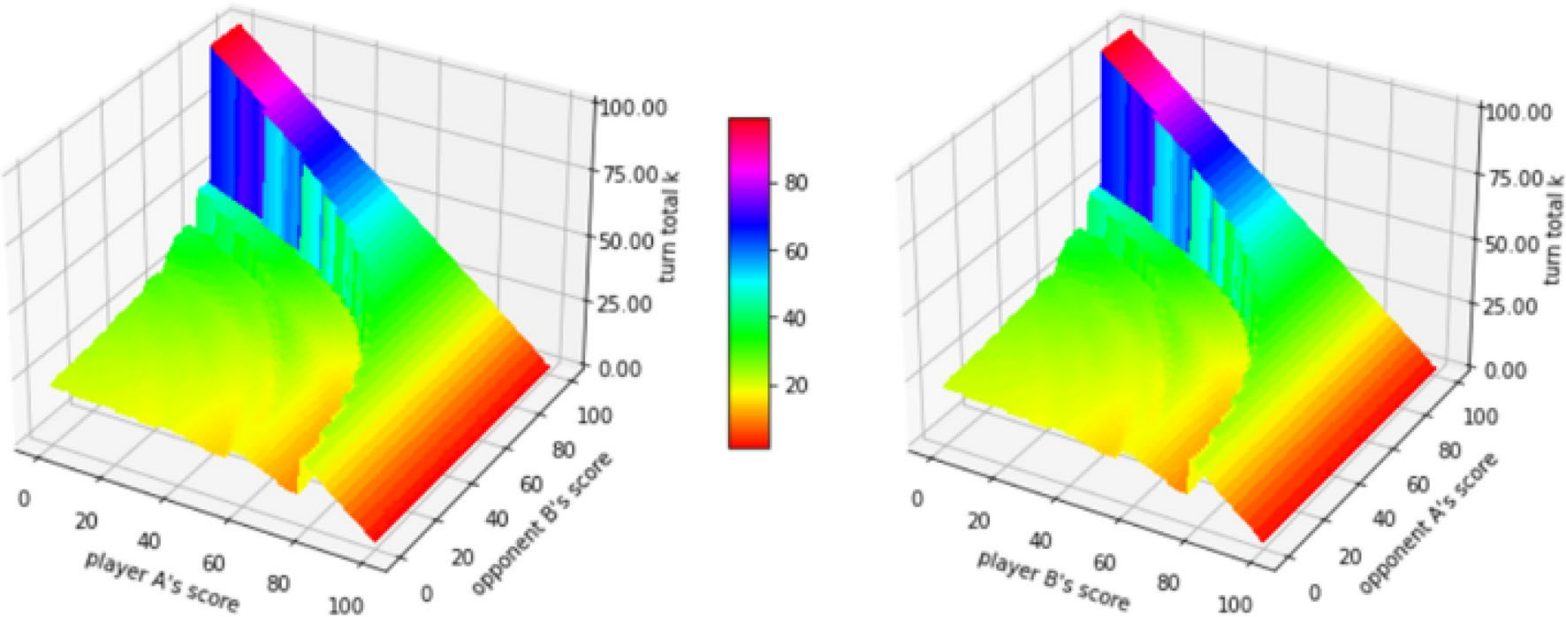
The contour surfaces of the Stackelberg equilibrium decisions. The left plot is the turn total *k* that player A should hold at for all states, and the right plot is the turn total *k* that player A should hold at for all states.

### Multiplayer simultaneous dice game

One advantage of the simultaneous Pig game is that it can be easily extended to multiple players, even with large number of players, without considering the order of play or decreasing players’ game experience. Another advantage is that the game is fair for every player, unlike in the sequential game where the playing order implies advantages or disadvantages towards winning. In the simultaneous game since every player begins their turns at the same time. There is no need to decide the order of play between the players. In this section, we shall first consider the 3-player simultaneous game and then extend which to infinite number of players.

#### Three players with independent strategies

Similar to the 2-player simultaneous game, here we have an additional player, denoted by the opponent player B2, with the first opponent player being B1. The state in the 3-player game becomes a 3-element tuple $(i,j_1,j_2)$ where *i* is player A’s entire score, $j_1$ and $j_2$ are the opponent players B1 and B2’s scores, respectively. Similarly, the action is still the value *k* that a player will hold at given a state $(i,j_1,j_2)$. Thus, the relationship between the state-value function *p*(*i*, *j*, *k*) and the action-value function $q(i,j_1,j_2,k)$ is:

18 $$\begin{aligned} \begin{aligned} p(i,j_1,j_2)&= \max _k q(i,j_1,j_2,k) \\ q(i,j_1,j_2,k)&= {{\,\mathrm{E}\,}}_{j_1',j_2'}\left[ p(i,j_1',j_2')P_{X_k}(0)+\sum _{r=0}^5p(i+r+k,j_1',j_2')P_{X_k}(k+r)\right] \end{aligned} \end{aligned}$$

where $j_1'$ and $j_2'$ are two random variables representing the entire scores of the opponent player B1 and B2 at the end of the turn, respectively. The distribution of $X_k$ can be found using the same approach introduced in the “Distribution calculation” section, as it is independent of the number of players and the players’ strategies. Since all players’ strategies are independent together per the nature of a simultaneous game, the value functions must satisfy the following symmetry property if the two opponent players B1 and B2 adopt the same strategy:

19 $$\begin{aligned} \begin{aligned} p(i,j_1,j_2)&= p(i,j_2,j_1) \\ q(i,j_1,j_2,k)&= q(i,j_2,j_1,k) \end{aligned} \end{aligned}$$

If all players’ strategies are independent, the corresponding optimal strategy $\pi _{A*}$ for player A given the opponent player B1’s strategy $\pi _{B1}$ and the opponent player B2’s strategy $\pi _{B2}$ can be found by Algorithm 0.3. Figure 5 shows the surface plot of the winning probability of the corresponding optimal strategy for player A, given different choices of the opponents’ strategy combinations. For example, if $\pi _{B1}=\pi _{B2}\equiv 25$, i.e. both two opponents adopt the “hold at 25” strategy, the winning probability of the corresponding optimal strategy for player A is 35.90%. If $\pi _{B1}\equiv 20$ and $\pi _{B2}\equiv 30$, the winning probability for player A adopting the corresponding optimal strategy increases to 39.55%. As the average winning rate for the 3-player game $\frac{1}{3}$ and “hold at 25” is the best simple strategy for the 2-player game, the corresponding optimal strategies found via value iteration can improve the winning rate decently compared to the tradition “hold at n” strategy. We exam different opponents’ strategy combinations with *n* ranges from 20 to 30. Not surprisingly, the best combination is that both opponents B1 and B2 adopt the “hold at 25” strategy given the 3-player game is independent, which is consistent to our previous result that “hold at 25” is the best simple strategy for the 2-player simultaneous game. The detailed winning probability of the corresponding optimal strategy against different combinations is shown in Supplementary Table S4.

**Figure 6:**
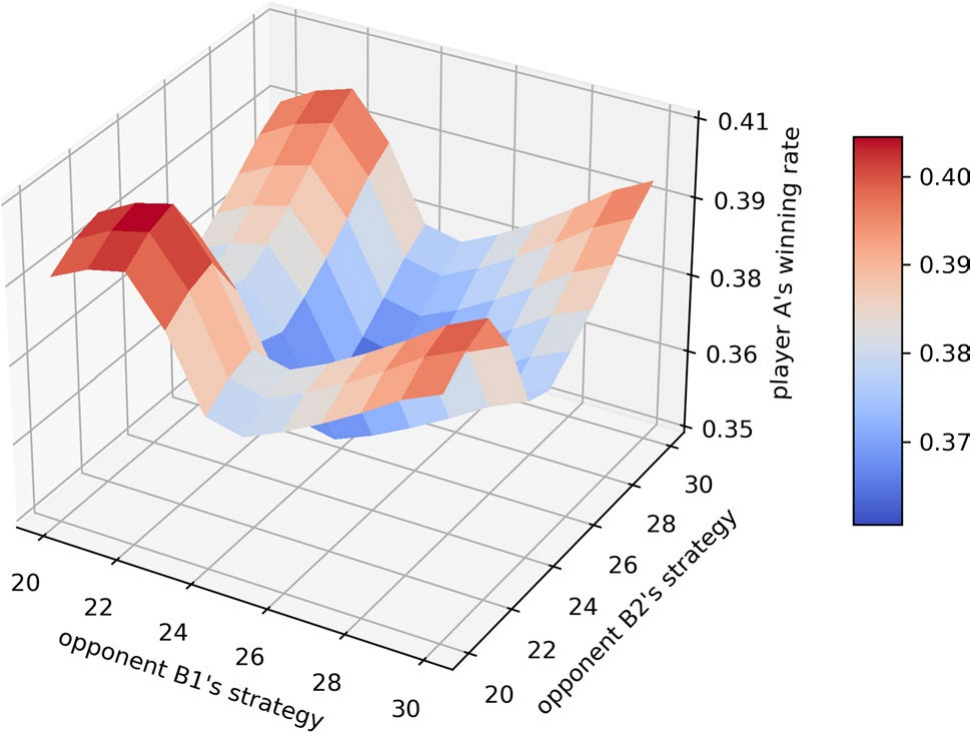
The surface plot of the winning probability of the corresponding optimal strategy for player A, given different opponents’ “hold at n” strategies. The *x*, *y* axes are the values *n* in the “hold at n” strategy for two opponents respectively.

For the 4-player simultaneous game, we can introduce another variable $j_3$ to record the score of the third opponent player, with the rest of the framework remains the similar. The overall logic flow for finite number of players adopting independent strategies is the same. As long as all players’ strategies remain independent, the value iteration can always find the corresponding optimal strategy for one player given the other players’ strategies. However, the computational costs increase as the number of players increases, with the computational complexity being $O(100^n)$, where *n* is the number of players.

#### Infinite number of players

When we further increase the number of players *n*, and even push *n* to infinity, what is the optimal strategy for each player? The limiting behavior of the optimal policy can be analyzed through symmetry. According to the simultaneous nature of the game, the Nash equilibrium or the optimal strategy is found when every player adopts the same strategy. The problem is then reduced to what is this strategy?

If every player uses the same strategy, the winning probability is the same for all players, namely $\frac{1}{n}$, where *n* is the number of players. For sufficiently large number of players, especially when $n \rightarrow {} \infty$, we know the expected winning chance for individual player is almost 0 as $\lim _{n \rightarrow \infty } \frac{1}{n} = 0$. However, based on the last row in Table 2, a player using the strategy “hold at 100” will have $1-0.9898=1.02\%$ chance to win the game in one turn. The overall winning chance for a single player adopting the “hold at 100” strategy is at least 1.02% given no other players adopting “hold at 100”. Thus, if every player adopts the same strategy which is not the “hold at 100” strategy, for a particular player A, he/she can easily switch the current strategy to the “hold at 100” strategy to increase his/her winning probability.

In generally, if there are *m* players selecting the “hold at 100” strategy, their expected individual winning probability is at least $\frac{1.02\%}{m}$. The probability that none of them reaches 100 in turn 1 is $(1-1.02\%)^m$. For the rest $n-m$ players not using the “hold at 100” strategy, they cannot win the game in turn 1 and thus their overall expected individual winning probability is at most $\frac{(1-1.02\%)^m}{n-m}$, which is close to 0 when *n* is sufficiently large, thus less than $\frac{1.02\%}{m}$ for any fixed *m*. Consequently, any player not adopting the “hold at 100” strategy will switch his/her strategy to the “hold at 100” strategy. Therefore, the Nash equilibrium is that every player will select the “hold at 100” strategy when there are infinite number of players.

In fact, when *n* is greater than or equal to 99, we have $\frac{1}{99} \approx 1.01\% < 1.02\%$. As long as the number of players exceed 98, then every player should only consider the “hold at 100” strategy.

## Discussion

The dice game Pig was originally designed for 2-player sequential play. In this work, we first discussed some potential issues of the current sequential Pig game and provided the simultaneous game as a solution. Then we thoroughly analyzed different cases of simultaneous Pig game, from independent strategy to optimal strategy, and from 2-player game to multiplayer game, using Markov decision process (MDP), dynamic programming (DP) and game theory, with the first two being classical components of reinforcement learning (RL). Before the reinforcement learning method is applied in the dice game Pig, the simple “hold at n” strategy is considered as a type of heuristic sub-optimal strategy with best the value n chosen through winning probabilities. This type of strategy is not dependent upon the opponent player’s score either. On the other hand, the optimal strategy developed by value iteration maximizes the winning probability at any state and is therefore a function of the opponent’s score, or even a function of the opponent’s strategy. This work has also demonstrated the power of RL in the field of games. In the sequential game, even the Nash equilibrium can be obtained through value iteration for the 2-player game (Neller and Presser^11^), but with some constraints for the 3-player game (Bonnet et al.^14^). While in the simultaneous game, value iteration does not converge for pure-strategy equilibrium, indicating only the mixed-strategy equilibrium which can be found by game theory exists. If sub-optimal is allowed, Stackelberg value iteration can be applied to find the pure strategy. However, in the case of independent strategies, value iteration can always find the corresponding optimal strategy for one player given the other players’ strategies, regardless of the number of players. In particular, we demonstrate how to use value iteration to find the corresponding optimal strategy for the 3-player simultaneous game. The result can be easily extended to any finite number of players. As for the limiting behavior in terms of the number of players, the pure-strategy Nash equilibrium is simply the “hold at 100” strategy, not surprisingly. Below we have summarized all the results based on related work in Table 4.

RL has sparked people’s imagination because the computer can learn on its own at lightning speed without training data from humans. The utility of RL goes well beyond providing the optimal play strategies for games. Rather, it has already rendered significantly impact in engineering (AI/robotics), finance, science, healthcare and so on, by greatly improving the framework for decision making in these fields. Because the pure-strategy equilibrium does not exist, value iteration encounters divergence, and the mixed-strategy equilibrium is obtained through game theory analysis. How to combine these two methods more tightly, i.e. to develop a RL algorithm that can directly find the mixed-strategy, will be our future focus. Furthermore, our work is based on a fixed player’s view, namely player A, and assumes no cooperation between the players. Using multi-agent reinforcement learning to incorporate the cooperative game is another direction worth future investigation.

**Table 4:** Summary of the dice game Pig.

Category	Two players		Three players	Infinite players
Subcategory	Independent strategy	Optimal strategy	Independent strategy	Optimal strategy
Sequential	Y	Y		X
Simultaneous	Y	Not convergent for pure strategy	Y	Y

Here character “Y” indicates the case is solved and the optimal strategy is found, while character “X” indicates that the case is unlikely. The independent strategy sequential game case is left blank as no related work was found. We believe this scenario is also solvable using value iteration.

## Supplementary Information

Supplementary Tables.

## Data Availability

Key data generated or analysed during this study are included in this published article and its supplementary information files. The rest datasets used and/or analysed during the current study (Fig. 2) available from the corresponding author on reasonable request.
