# Supplementary material for: Optimal strategy of the simultaneous dice game Pig for multiplayers: when reinforcement learning meets game theory
Source: Sci Rep. 2023 May 19;13:8142. doi: 10.1038/s41598-023-35237-x (PMC10199029; doi:10.1038/s41598-023-35237-x)
Supplement: Supplementary file 1 — Supplementary Tables. [file 41598_2023_35237_MOESM1_ESM.pdf]

Supplementary Material

Table S1: Given different "hold at n" strategies, the winning probability of the corresponding optimal strategy. The n ranges from 10 to 50 with additional case $n = 100$. The best scenario is $n = 25$, as the winning probability for the corresponding optimal strategy is the lowest.

Opponent Hold at n	Winning Prob for Corresponding Optimal Strategy
10	0.739274
11	0.700797
12	0.674509
13	0.638227
14	0.629074
15	0.602079
16	0.581321
17	0.585311
18	0.570558
19	0.543235
20	0.543684
21	0.552774
22	0.559380
23	0.547593
24	0.524985
25	0.523147
26	0.530766
27	0.540349
28	0.551323
29	0.563247
30	0.573066
31	0.571347
32	0.556559
33	0.547559
34	0.550053
35	0.554804
36	0.561083
37	0.568799
38	0.577715
39	0.587599
40	0.598287
41	0.609584
42	0.621270
43	0.633246
44	0.645442
45	0.657702
46	0.668586
47	0.674880

Continue on the next page

Opponent Hold at n	Winning Prob for Corresponding Optimal Strategy
48	0.673680
49	0.668771
50	0.665939
100	0.884749

Table S2: Part of the corresponding optimal strategy against the "hold at 25" strategy, as shown in Figure 3. The actions for the player given different opponent's scores (0, 25, 50), when the opponent uses the "hold at 25" strategy.

Player's Score	Opponent's Score = 0	Opponent's Score = 25	Opponent's Score = 50
0	21	23	29
1	21	23	29
2	21	23	29
3	21	23	29
4	20	23	29
5	20	23	28
6	20	23	28
7	20	23	27
8	20	23	27
9	20	23	26
10	20	23	26
11	20	23	25
12	20	22	25
13	20	22	24
14	20	22	25
15	20	22	27
16	20	22	27
17	20	21	26
18	19	22	26
19	19	22	26
20	19	22	26
21	19	22	26
22	19	22	26
23	19	22	25
24	19	22	25
25	19	21	26
26	19	21	26
27	18	21	26
28	18	21	26
29	18	20	26
30	17	20	25
31	17	20	25
32	17	20	25
33	17	20	24

Continue on the next page

Player's Score	Opponent's Score = 0	Opponent's Score = 25	Opponent's Score = 50
34	17	20	24
35	18	20	24
36	18	20	24
37	18	20	23
38	18	19	23
39	18	19	23
40	18	19	22
41	18	19	22
42	17	18	27
43	17	18	27
44	17	18	26
45	17	18	25
46	16	18	24
47	16	19	24
48	16	19	23
49	15	19	22
50	15	19	21
51	14	18	21
52	14	17	20
53	14	17	19
54	14	16	18
55	14	16	18
56	16	20	17
57	17	20	16
58	17	19	15
59	17	19	15
60	16	18	15
61	16	17	15
62	17	17	14
63	16	16	14
64	16	15	14
65	15	14	14
66	15	14	14
67	14	13	14
68	13	12	14
69	13	11	31
70	12	11	30
71	11	10	29
72	10	9	28
73	10	9	27
74	9	9	26
75	8	10	25
76	8	24	24
77	7	23	23
78	22	22	22

Continue on the next page

Player's Score	Opponent's Score = 0	Opponent's Score = 25	Opponent's Score = 50
79	21	21	21
80	20	20	20
81	19	19	19
82	18	18	18
83	17	17	17
84	16	16	16
85	15	15	15
86	14	14	14
87	13	13	13
88	12	12	12
89	11	11	11
90	10	10	10
91	9	9	9
92	8	8	8
93	7	7	7
94	6	6	6
95	5	5	5
96	4	4	4
97	3	3	3
98	2	2	2
99	1	1	1

Table S3: The detailed states that the two policies found by Stackelberg Value Iteration have different values. Both Player's Score and Opponent's Score are scores at the beginning of a turn. The values for columns "Policy π_A" and "Policy π_B" are the actions for the corresponding state (Player's Score, Opponent's Score). For policy π_A, Player's Score means A's score and Opponent's Score means B's score. While for policy π_B, Player's Score means B's score and Opponent's Score means A's score.

	Player's Score	Opponent's Score	Policy π_A	Policy π_B
1	2	60	33	34
2	4	54	28	29
3	6	53	28	29
4	8	1	21	20
5	8	34	24	25
6	8	53	28	29
7	8	57	30	31
8	9	29	24	23
9	9	34	25	24
10	11	57	30	31
11	11	58	32	33
12	12	57	30	31
13	13	53	27	28
14	14	56	29	30
15	14	57	31	30

Continue on the next page

	Player's Score	Opponent's Score	Policy π_A	Policy π_B
16	16	70	29	28
17	17	55	28	29
18	19	52	26	25
19	20	52	26	25
20	20	75	40	39
21	21	52	26	25
22	21	54	28	27
23	22	52	26	25
24	22	69	37	36
25	23	53	27	28
26	24	45	25	24
27	24	51	25	26
28	26	52	27	26
29	27	15	21	20
30	27	50	25	26
31	28	51	26	27
32	29	48	24	23
33	30	49	25	24
34	33	46	23	24
35	34	7	20	19
36	35	45	23	24
37	36	43	22	23
38	43	37	20	21
39	44	34	20	21
40	44	70	28	29
41	47	26	20	19
42	47	30	20	19
43	47	31	19	20
44	48	28	20	19
45	48	32	19	18
46	48	38	24	20
47	49	16	17	18
48	50	27	19	18
49	51	10	17	16
50	52	10	18	17
51	52	25	18	17
52	52	30	18	22
53	52	67	24	23
54	53	4	15	16
55	53	29	22	21
56	54	20	18	17
57	54	25	17	21
58	54	66	23	22
59	55	18	17	18
60	55	27	21	20

Continue on the next page

	Player's Score	Opponent's Score	Policy π_A	Policy π_B
61	56	15	17	18
62	56	16	17	18
63	56	65	22	21
64	57	6	18	17
65	57	7	18	17
66	57	13	17	18
67	58	12	18	17
68	59	63	21	22
69	60	10	17	18
70	62	8	17	16
71	63	59	20	37
72	64	6	15	16
73	69	47	31	16
74	73	32	13	27

Table S4: The winning probability of the corresponding optimal strategy for player A against different "hold at n" strategy combinations for the opponent players B1 and B2, where n ranges from 20 to 30, with row being B1's strategy and column being B2's strategy. The probability matrix is symmetric and the minimum winning probability for player A happens when both opponents B1 and B2 adopt the "hold at 25" strategy.

Hold at	20	21	22	23	24	25	26	27	28	29	30
20	0.3958	0.3992	0.4008	0.3921	0.3782	0.3765	0.3797	0.3835	0.3877	0.3923	0.3955
21	0.3992	0.4028	0.4045	0.3957	0.3815	0.3798	0.3831	0.3870	0.3914	0.3962	0.3995
22	0.4008	0.4045	0.4063	0.3975	0.3832	0.3815	0.3848	0.3889	0.3934	0.3983	0.4017
23	0.3921	0.3957	0.3975	0.3889	0.3752	0.3735	0.3767	0.3806	0.3850	0.3898	0.3931
24	0.3782	0.3815	0.3832	0.3752	0.3622	0.3606	0.3636	0.3673	0.3714	0.3758	0.3790
25	0.3765	0.3798	0.3815	0.3735	0.3606	0.3590	0.3620	0.3657	0.3698	0.3742	0.3774
26	0.3797	0.3831	0.3848	0.3767	0.3636	0.3620	0.3651	0.3689	0.3731	0.3777	0.3809
27	0.3835	0.3870	0.3889	0.3806	0.3673	0.3657	0.3689	0.3728	0.3772	0.3819	0.3852
28	0.3877	0.3914	0.3934	0.3850	0.3714	0.3698	0.3731	0.3772	0.3817	0.3866	0.3901
29	0.3923	0.3962	0.3983	0.3898	0.3758	0.3742	0.3777	0.3819	0.3866	0.3917	0.3954
30	0.3955	0.3995	0.4017	0.3931	0.3790	0.3774	0.3809	0.3852	0.3901	0.3954	0.3992
